# Thyroid malignancy among patients with thyroid nodules in the United Arab Emirates: a five-year retrospective tertiary Centre analysis

**DOI:** 10.1186/s13044-018-0061-x

**Published:** 2018-11-30

**Authors:** Eiman Alseddeeqi, Raqwana Baharoon, Rawia Mohamed, Jenan Ghaith, Abeer Al-Helali, Luai A. Ahmed

**Affiliations:** 1 Division of Endocrinology, Sheikh Khalifa Medical City, P.O. Box 51900, Abu Dhabi, UAE; 2 Division of Nuclear Medicine, Sheikh Khalifa Medical City, Abu Dhabi, UAE; 3 Division of Pathology, Sheikh Khalifa Medical City, Abu Dhabi, UAE; 4 Division of Internal Medicine, Sheikh Khalifa Medical City, Abu Dhabi, UAE; 5 Division of Radiology, Sheikh Khalifa Medical City, Abu Dhabi, UAE; 6 Institute of Public Health, College of Medicine and Health Sciences, United Arab Emirates University, Al Ain, UAE

**Keywords:** Thyroid, Thyroid nodules, Thyroid Cancer, Cancer, Prevalence, United Arab Emirates

## Abstract

**Objectives:**

Thyroid malignancy constitutes the sixth common cancer type in the United Arab Emirates (UAE). There are no epidemiological data outlining the prevalence of cancer in thyroid nodules, nor previous analysis of ultra-sonographic features correlating with thyroid malignancy in the UAE. This study aimed to estimate the prevalence of thyroid malignancy in patients with thyroid nodules and to describe the ultra-sonographic characteristics of thyroid nodules harbouring malignancy.

**Methods:**

A retrospective electronic medical records review of all thyroid nodules in patients (aged 18 to 80 years) with normal thyroid-stimulating hormone (TSH) levels, who underwent ultrasound guided fine needle aspiration cytology (UG-FNA) at Sheikh Khalifa Medical City (SKMC) during 2011–2015.

**Results:**

436 patients with normal TSH underwent UG-FNA cytological examination of thyroid nodules (*n* = 555 nodules). The overall crude prevalence of thyroid cancer among patients was 10.1% (95% CI 7.5–13.3). The age-adjusted prevalence of thyroid cancer among UAE nationals, Arabs, Far East Asians, and Caucasians were 9.6% (3.6–15.6), 10.0% (6.2–13.8), 16.8% (4.5–29.0) and 16.3% (1.7–30.9), respectively. The crude prevalence was 14.5%(95% CI 6.2–22.8) in men, and 9.3%(95% CI 6.3–12.2) in women. The echogenicity features were significantly different between the cancerous and noncancerous nodules (*p* = 0.025). Cancerous nodules were relatively more hyper- and hypo-echoic, while noncancerous nodules were mostly complex.

**Conclusion:**

We report a higher prevalence of thyroid malignancy among patients with thyroid nodules relative to that reported in other parts of the world. The rate of thyroid malignancy was higher in patients of Far-East Asian and Caucasian ethnic background.

## Introduction

The overall worldwide incidence of thyroid malignancy has been increasing over the last four decades [1]. Ongoing research is aimed at exploring possible factors contributing to the formation of different thyroid cancer subtypes, to identify individuals at risk of thyroid malignancy [2].

Thyroid nodules are another common thyroid endocrinopathy with prevalence ranging from 1 to 5% in iodine-sufficient areas; this prevalence is significantly increased if nodules incidentally detected by ultrasound are included, reaching up to 68% [3]. Moreover, the overall number of thyroid nodules and malignancy cases reported in the last decade has increased due to widespread use of neck ultrasound [3].

The United Arab Emirates (UAE) is classified as an area with minimal iodine-deficiency with rate of households using iodized salt around 6% and a median urinary iodine excretion of 100 μg/l. Therefore, a high prevalence of thyroid malignancy is not unexpected [4–7]. Thyroid malignancy is the most common endocrine malignancy in the UAE, and papillary thyroid cancer is the most common type of thyroid cancer [4]. It is reported as the sixth most common cancer type among UAE population and the second among UAE women [4].

There are no comprehensive epidemiological data outlining the prevalence of thyroid malignancy in thyroid nodules and its correlation with demographic data in UAE population (a heterogeneous population with multiple ethnic backgrounds). Moreover, there is no previous analysis of ultra-sonographic features correlating with thyroid malignant nodules in the UAE.

This study aimed to describe the prevalence of thyroid malignancy in euthyroid patients with thyroid nodules in the UAE population. The study also aims to analyse the distribution of thyroid malignancy by gender, age, ethnicity, cytological, and ultra-sonographic characteristics.

## Methods

### Study design and settings

This is a retrospective analysis of a cohort of patients (aged 18 years old and above with thyroid nodules and normal serum TSH) who underwent ultrasound guided fine needle aspiration (UG-FNA) cytology for the first time from January 2011 to December 2015, at Sheikh Khalifa Medical City (SKMC) in Abu Dhabi. SKMC is a tertiary centre and functioned as the referral centre for peripheral health care facilities during the study period. The normal reference range for TSH is 0.270–4.200 mIU/L and all TSH levels were measured at our institution. Neck ultrasound and reporting were performed by more than one radiologist while decision to perform aspiration cytology was made by endocrinologists based on ultrasound findings. The diagnosis of cancer was made following surgery by one experienced certified pathologist per each case. If features of malignancy are overlapping with benignity on histology specimens, a second opinion is sought through the monthly pathology meeting dedicated for such cases.

An institutional research board approval was obtained from SKMC.

All patients with known thyroid cancer history and abnormal TSH level were excluded.

### Chart review data

Demographic characteristics of all patients who underwent cytological examinations were retrieved from their medical records. These included, age at the time of performing the fine needle aspiration, gender and ethnicity. Age was grouped as follows: 18–29, 30–39, 40–49, 50–59 and ≥ 60 years. Ethnicity was classified according to nationalities into six major groups: UAE nationals, Arabs (except UAE), Far-East Asians (Philippines, China), South-East Asians (India, Pakistan, Bangladesh, and Sri Lanka), Caucasians, and others.

UG-FNA was performed on all nodules sized ≥1 cm if they demonstrated worrisome ultra-sonographic features; and ultrasound features were reported according to the American Thyroid Association (ATA) clinical practice guidelines for assessment of thyroid nodule (2009) [8].

In case of a patient with more than one nodule, criteria for FNA selection were based on the size and presence of a worrisome feature on ultrasound as mentioned above. For each nodule, the size (in three dimensions with the greatest dimension used for data analysis) and echogenicity features (solid, cystic, or complex) were identified. For solid nodules, echogenicity was classified into either: is-echogenicity, hypo-echogenicity, or hyper-echogenicity. Other ultrasound features reported included the presence of nodal disease or calcifications.

Bethesda thyroid nodule cytological classification reporting system was used to report the cytopathology [9]. Nodules with Bethesda categories IV, V, and VI underwent lobectomy or total thyroidectomy with histological examination. For Bethesda III cytology result, a repeat fine needle aspiration cytology or lobectomy was recommended based on the presence of worrisome features on ultrasound. Thyroid malignancy was divided into subtypes as follows: classical papillary thyroid cancer (CPTC), follicular variant of papillary thyroid cancer (FvPTC), classical follicular thyroid cancer (FTC), Hurthle thyroid cancer and medullary thyroid cancer (MTC).

### Statistical analysis

Data were analysed as per-patient and per-nodule basis. Demographic of the patients with nodules and the ultra-sonographic and cytological characteristics of the nodules were described. Categorical data were expressed as numbers and percentages. Continuous data were reported as means and standard deviations. Chi-squared or Fisher’s tests were used to determine the statistical significance of the differences between the characteristics, when comparing cancerous and noncancerous nodules. The overall and gender-specific prevalence of cancerous nodules by demographic characteristics were estimated using logistic models and reported with 95% confidence intervals (CI). Stata 15 was used to perform the analysis. The significance level of the statistical tests was set at 5%.

## Results

### Demographics

**Fig. 1:**
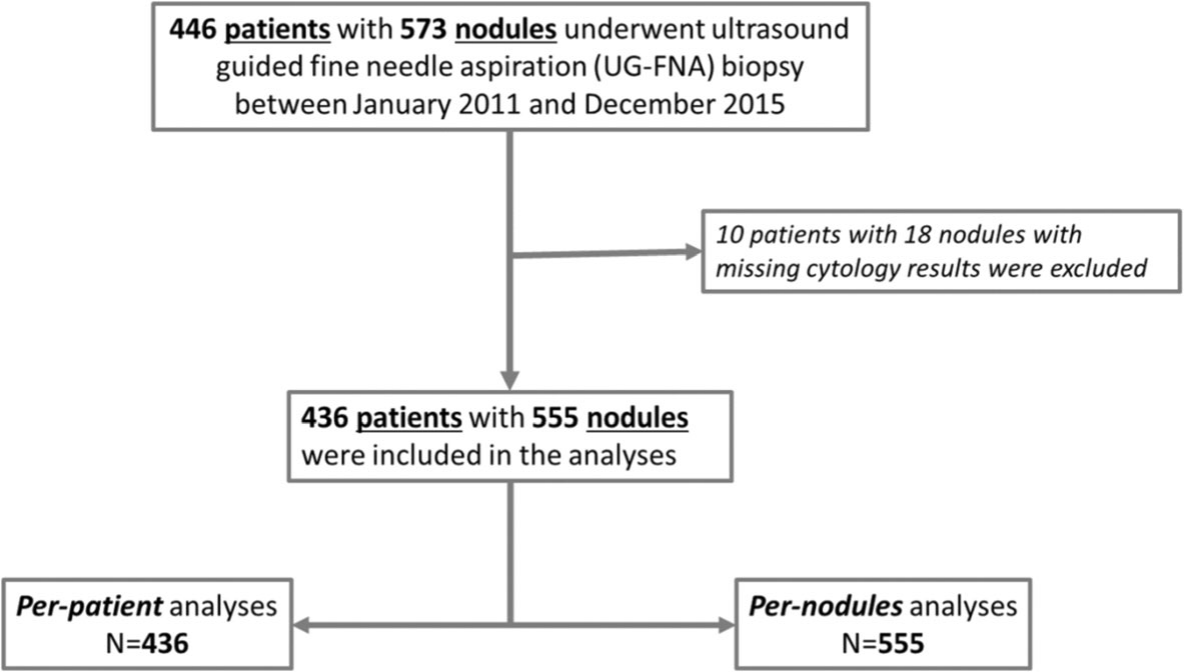
Numbers of patients and thyroid nodules included in the analyses

**Fig. 2:**
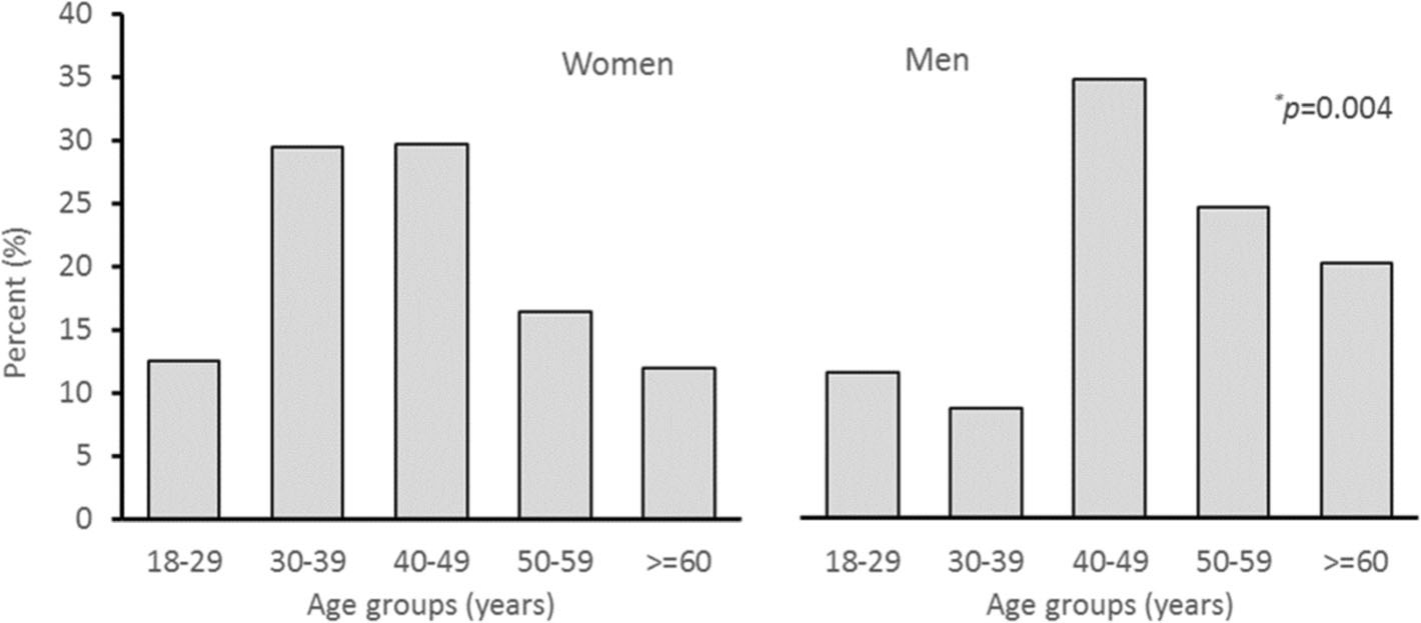
Age and gender distribution of patients with thyroid nodules (*N* = 555^*^) who underwent ultrasound guided fine needle aspiration (UG-FNA) cytology between January 2011 and December 2015 in a tertiary hospital in Abu Dhabi, United Arab Emirates (^*^471 nodules in women and 84 nodules in men)

Figure 1 shows the included numbers of patients and nodules in the analyses. 436 patients (367 women, 69 men) with normal TSH were included in the study. Table 1 shows the general characteristics of all patients. The overall mean age was 44.1 years (standard deviation (SD) 12.7 years). The mean (SD) age in women and men were 43.4 (12.7) and 47.8 (12.2), respectively (*p* = 0.008). Moreover, there were significant differences in the distribution of gender by age-groups (*p* = 0.004). 59.2% of women aged between 30 and 49 years whereas 58.3% of men aged between 40 and 59 years as illustrated in Fig. 2. Around 77% of the patients were of Arabic ethnicity (including UAE nationals).

**Table 1:** Characteristics of patients with thyroid nodules (*N* = 436) that underwent ultrasound guided fine needle aspiration (UG-FNA) cytology between January 2011 and December 2015 in a tertiary hospital in Abu Dhabi, United Arab Emirates

	Number *(%)*
N	
Gender	
*Women*	*367 (84.2)*
*Men*	*69 (15.8)*
Age (*years)* mean (SD)	*44.1 (12.7)*
Age groups	
*18–29 years*	*54 (12.4)*
*30–39 years*	*114 (26.2)*
*40–49 years*	*133 (30.5)*
*50–59 years*	*77 (17.7)*
*≥ 60 years*	*58 (13.3)*
Ethnicity^a^	
*UAE*	*95 (21.4)*
*Arabs*	*241 (55.4)*
*Far-East Asians*	*36 (8.3)*
*South-East Asians*	*29 (6.7)*
*Caucasians*	*26 (6.0)*
*Others*	*8 (1.8)*

^a^One patient with missing ethnicity

### Prevalence

The overall crude prevalence of thyroid cancer in patients with thyroid nodules was 10.1% (95% CI 7.5–13.3). The crude prevalence was 9.3% (95% CI 6.3–12.2) in women, and 14.5% (95% CI 6.2–22.8) in men (Table 2). Similar to the crude prevalence, the overall age-adjusted prevalence was 10.0% (7.7–12.8), and 9.0% (6.0–11.9) in women; whereas slightly higher in men (15.1% (6.5–23.8)). Overall, there was no significant difference in age-adjusted thyroid cancer prevalence between women and men (*p* = 0.117).

**Table 2:** Crude and age-adjusted overall and gender-specific thyroid cancer prevalence (95% CI) among patients with thyroid nodules who underwent ultrasound guided fine needle aspiration (UG-FNA) biopsy between January 2011 and December 2015 in a tertiary hospital in Abu Dhabi, United Arab Emirates

	Crude Cancer Prevalence *% (95% CI)*			
	Overall	Women	Men	*p*-value^*^
N (cancer/No cancer)	44/392	34/333	10/59	
All thyroid nodules	10.1 (7.5–13.3)	9.3 (6.3–12.2)	14.5 (6.2–22.8)	*0.186*
Age (years)				
*18–29 years*	16.7 (6.7–26.6)	15.2 (4.8–25.6)	25.0 (0.0–55.0)	*0.493*
*30–39 years*	8.8 (3.6–14.0)	8.3 (3.1–13.5)	16.7 (0.0–46.5)	*0.482*
*40–49 years*	12.0 (6.5–17.6)	11.0 (5.1–16.9)	16.7 (1.8–31.6)	*0.441*
*50–59 years*	5.2 (0.2–10.2)	5.0 (0.0–10.5)	5.9 (0.0–17.1)	*0.885*
*≥ 60 years*	8.6 (1.4–15.8)	6.8 (0.0–14.3)	14.3 (0.0–32.6)	*0.386*
Ethnicity^**^				
*UAE*	9.5 (3.6–15.4)	7.7 (1.8–13.6)	17.6 (0.0–35.8)	*0.204*
*Arabs*	10.4 (6.5–14.2)	*10.1 (5.9–14.3)*	*11.6 (2.0–21.2)*	*0.766*
*Far-East Asians*	16.7 (4.5–28.8)	*12.1 (1.0–23.3)*	*66.7 (13.3–99.9)*	*0.015*
*Caucasians*	15.4 (1.5–29.3)	*16.0 (1.6–30.4)*	*0*	
	Age-adjusted Cancer Prevalence *% (95% CI)*			
All thyroid nodules	10.0 (7.7–12.8)	9.0 (6.0–11.9)	15.1 (6.5–23.8)	*0.117*
Ethnicity^a^				
*UAE*	9.6 (3.6–15.6)	7.7 (1.8–13.7)	18.7 (0.0–37.9)	*0.166*
*Arabs*	10.0 (6.2–13.8)	*9.7 (5.5–13.8)*	*10.9 (1.5–20.4)*	*0.799*
*Far-East Asians*	16.8 (4.5–29.0)	*12.3 (1.0–23.6)*	*65.1 (10.4–99.9)*	*0.019*
*Caucasians*	16.3 (1.7–30.9)	*17.0 (1.8–32.1)*	*0*	

^*^p-value comparing women vs men

^a^No cancerous nodules in patients of South-East Asians/Other ethnicities

The age-adjusted prevalence of thyroid cancer among UAE nationals, Arabs, Far East Asians, and Caucasians were 9.6% (3.6–15.6), 10.0% (6.2–13.8), 16.8% (4.5–29.0) and 16.3% (1.7–30.9), respectively; with relatively higher cancer prevalence in men. Only for Far-East Asians patients, the cancer prevalence was significantly higher in men compared to women (*p* = 0.019). No cancer was reported in thyroid nodules detected in South-East Asian or ‘Other’ ethnicities’ patients.

### Ultra-sonographic and cytological characteristics of the nodules

In total of 555 thyroid nodules underwent UG-FNA cytological examinations. As Table 3 shows, more than 60% of the nodules were sized 2 cm or more with no difference in the size distribution between the cancerous and noncancerous nodules (*p* = 0.176). Around 54% of the nodules were of complex echogenicity. The echogenicity features were significantly different between the cancerous and noncancerous nodules (*p* = 0.025). Cancerous nodules were relatively more hyper- and hypo-echoic, while noncancerous nodules were mostly complex. Although 70% of the nodules had no calcifications, coarse calcification occurred more frequently with cancerous nodules compared to noncancerous nodules (*p* < 0.001). Around 79% of all the nodules were classified as Bethesda II while cancerous nodules were more frequently classified as Bethesda IV, V, or VI compared to noncancerous nodules (*p* < 0.001).

**Table 3:** Characteristics of thyroid nodules (*N* = 555) that underwent ultrasound guided fine needle aspiration (UG-FNA) biopsy between January 2011 and December 2015 in a tertiary hospital in Abu Dhabi, United Arab Emirates

	Total N (%^*^)	Cancer status		*p*-value^**^
		Cancer N (%^a^)	No cancer N (%^a^)	
N	555 (100)	49 (100)	506 (100)	
Nodule size	535 (100)	48 (100.0)	487 (100.0)	0.176
*< 1 cm*	*27 (5.1)*	*5 (10.4)*	*22 (4.5)*	
*1 – < 2 cm*	*174 (32.5)*	*17 (35.4)*	*157 (32.2)*	
*2–4 cm*	*222 (41.5)*	*20 (41.7)*	*202 (41.5)*	
*> 4 cm*	*112 (20.9)*	*6 (12.5)*	*106 (21.8)*	
Echogenicity	531 (100)	47 (100.0)	484 (100.0)	0.025
*Cystic*	*15 (2.8)*	*1 (2.1)*	*14 (2.9)*	
*Complex*	*287 (54.1)*	*17 (36.2)*	*270 (55.8)*	
*Hyper-echoic*	*18 (3.4)*	*4 (8.5)*	*14 (2.9)*	
*Hypo-echoic*	*124 (23.4)*	*17 (36.2)*	*107 (22.1)*	
*Isoechoic*	*87 (16.4)*	*8 (17.0)*	*79 (16.3)*	
Calcification	547 (100)	48 (100.0)	499 (100.0)	< 0.001
*Coarse*	*25 (4.6)*	*14 (29.2)*	*11 (2.2)*	
*Yes*	*125 (22.9)*	*10 (20.8)*	*115 (23.0)*	
*No*	*397 (72.6)*	*24 (50.0)*	*373 (74.8)*	
Lymphadenopathy	554 (100)	49 (100)	505 (100)	0.999
*No*	*530 (95.7)*	*47 (95.9)*	*483 (95.6)*	
*Yes*	*24 (4.3)*	*2 (4.1)*	*22 (4.4)*	
FNA Cytology	551 (100)	49 (100)	502 (100)	< 0.001
*Bethesda, I*	*29 (5.3)*	*1 (2.0)*	*28 (5.6)*	
*Bethesda II*	*435 (79.0)*	*4 (8.2)*	*431 (85.9)*	
*Bethesda III*	*32 (5.8)*	*6 (12.2)*	*26 (5.2)*	
*Bethesda IV*	*25 (4.5)*	*17 (34.7)*	*8 (1.6)*	
*Bethesda V*	*16 (2.9)*	*12 (24.5)*	*4 (0.8)*	
*Bethesda VI*	*14 (2.5)*	*9 (18.4)*	*5 (1.0)*	

^a^Column percent. ^**^p-value comparing cancerous vs noncancerous groups

(*20, 24, 8, 1, & 4 nodules were missing size, echogenicity, calcification, lymphadenopathy, & FNA cytology information, respectively*)

**Fig. 3:**
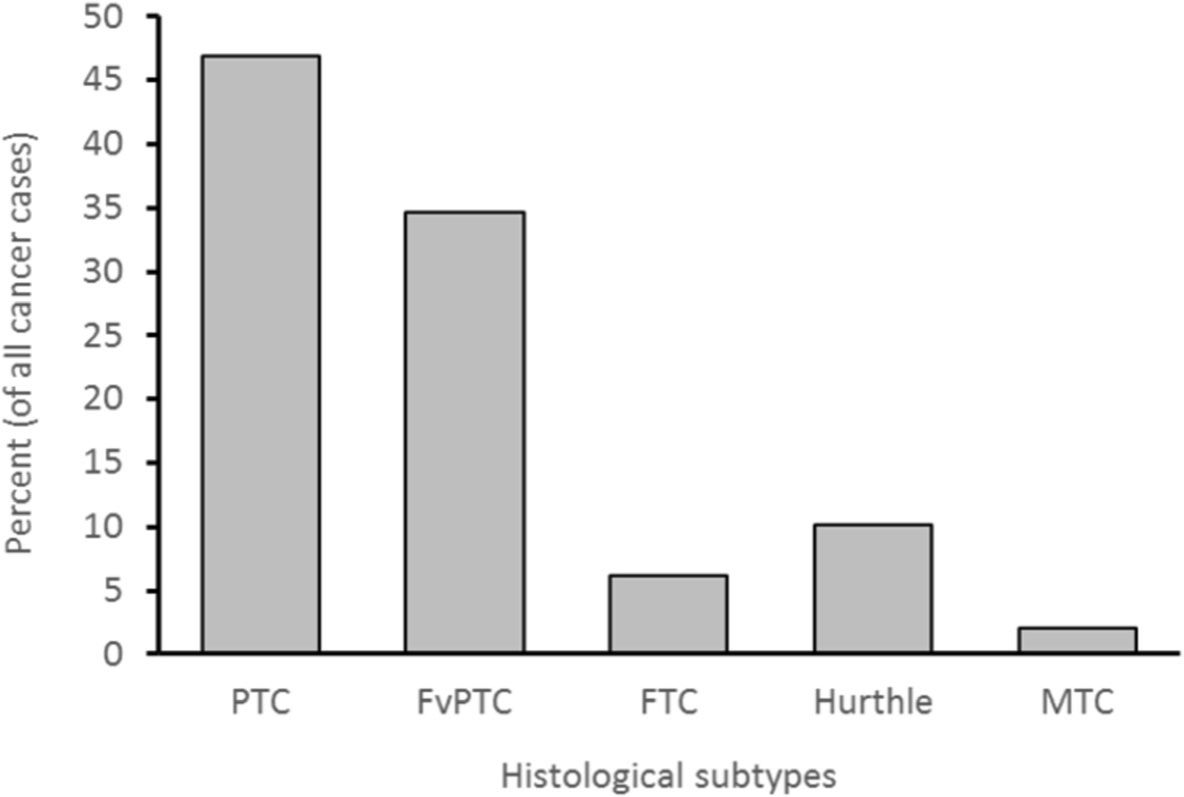
Distribution of histological subtypes of cancerous thyroid nodules (*N* = 49) underwent ultrasound guided fine needle aspiration (UG-FNA) cytology between January 2011 and December 2015 in a tertiary hospital in Abu Dhabi, United Arab Emirates. *(PTC: Papillary Thyroid cancer, FvPTC: Follicular Variant of Papillary Thyroid Cancer, FTC: Follicular Thyroid Cancer, MTC: Medullary Thyroid Cancer)*

Among the cancerous thyroid nodules, 47% were PTC while 35% were FvPTC (Fig. 3). No significant histological differences were found between women and men in cancerous nodules (*p* = 0.443).

## Discussion

This study is the first to estimate the prevalence of thyroid cancer among thyroid nodules in the UAE and covered the period from 2011 to 2015. Relatively high prevalence ranging between 9 to 15% were found in women and men. High malignancy rates were found in nodules with sizes between 2 and 4 cm.

The overall cancer crude prevalence of 10.1% which was found among thyroid nodules in our study is relatively high compared to the prevalence reported in some Western countries. The prevalence of thyroid malignancy in a single nodule in a study performed in the United States was 5% [6]. Finding of a similar study conducted in Italy supported the same lower prevalence (3.3%) of thyroid malignancy among thyroid nodules [6]. A study by Santos J. et al. found an association between increased risk of thyroid nodules and thyroid malignancy in iodine-deficient areas [5]. Another regional study reported the same association of increased malignant thyroid nodules among iodine-deficient subjects [10]. This could explain the relatively higher malignancy formation in thyroid nodules found in our study as most of the study population descend from iodine deficient parts of the world. However, the rate found in our study was more or less similar to other reported prevalence in Saudi Arabia, Cyprus, and Taiwan, where thyroid cancer rates among thyroid nodules ranged from 7 to 15% [11–13].

We found an 10.0% overall age-adjusted prevalence of thyroid cancer; with 9.0% in women and 15.1% in men. This gender differences with higher rate in men is consistent with findings from an Asian population study [8]. In this study, although the highest proportions of thyroid and cancerous nodules occurred among subjects aged 40–49 years, thyroid malignancy prevalence was highest among thyroid nodules in women aged 18–29 years and men aged 30–39 years. An increased unmet demand of iodine during pregnancy in the reproductive age group may explain the increased frequency of thyroid nodules with a younger age presentation in women; and hence, the associated occurrence of malignancy in women compared to men [5, 14, 15]. Another possible factor for the earlier presentation in women during the childbearing years is the associated level of oestrogen which is known to induce thyroid cancer in mice [16].

Interestingly, the prevalence of thyroid malignancy in this study varies between patients of different ethnic backgrounds. It was found to be highest among Far-East Asians and among Caucasian populations presenting with thyroid nodules. Higher rates of thyroid malignancy were reported previously among Far-East Asians in North America [17]. A similar study was performed in North America where it was found that thyroid cancers were proportionally higher in both foreign- and United States-born Arabs compared with other metropolitan ethnicities [18]. Further research to identify risk factors influencing malignancy occurrence in certain ethnic groups is warranted. Ultrasound examination is readily available in the UAE in secondary care centres; therefore, the increased detection rate of thyroid nodules by ultrasound might have contributed to the increased prevalence of thyroid malignancy in this study. Therefore, we speculate that ethnicity, iodine-deficiency (which leads to the formation of multinodular goiters), and the wide spread use of ultrasound, led to the detection of relatively higher prevalence of thyroid cancer in our study.

Most nodules in this study were classified as Bethesda II cytology type (76.6%), in which thyroid malignancy is not common. Conventional PTC was found in 46.9% of all cancer types while FvPTC constituted 34.7% of all thyroid malignancies in our study. This result is consistent with reports from different parts of the world that PTC constitutes 80–90% of all thyroid malignancy types [1, 19]. In terms of ultrasound characteristics, around 41.3% of nodules with a size of between 2 and 4 cm was associated with the occurrence of thyroid malignancy in our study. This result is consistence with other studies demonstrating similar association with size [20, 21]. Even the most recent ATA guidelines (2015) showed that the potential of malignancy in nodules < 2 cm is low in the absence of other worrisome features [22]. Therefore, FNA of thyroid nodules is indicated for all thyroid nodules above 2 cm. An exception to that is a nodule that is purely cystic or that has a spongiform pattern on ultrasound [22]. The presence of such ultra-sonographic worrisome features could indicate the need to decrease the threshold required for performing thyroid nodule FNA, especially in certain ethnic groups or in all ethnicities when certain size criteria is met.

### Limitations and strengths

This retrospective data analysis occurred in a single centre. The analysis was performed on nodules managed according to the ATA 2009 standard of practice guidelines for managing thyroid nodules. Referrals to our tertiary centre could have created a referral bias, however, FNA indication was based on certain clinical criteria. Since histological confirmation was not performed on all thyroid nodules, the rates of thyroid malignancy could have been underestimated. Among the strength of this study is the large sample size of patients and nodules included. Moreover, all patients included in the study had normal TSH to ensure validity of the results because patients from iodine-deficient areas exhibit more autoimmune thyroid conditions as well as increased risk of thyroid malignancy when presenting with high TSH [14].

## Conclusion

The prevalence of thyroid malignancy in this study is higher relative to that reported in other parts of the world. Far-East Asian and Caucasian ethnic groups were found to have higher rates of thyroid malignancy.

## Abbreviations

ATA: American Thyroid Association
CPTC: Conventional papillary thyroid cancer
FTC: Classical follicular thyroid cancer
FvPTC: Follicular variant of papillary thyroid cancer
MTC: Medullary thyroid cancer
SKMC: Sheikh Khalifa Medical City
TSH: Thyroid-stimulating hormone
UAE: United Arab Emirates
UG-FNA: Ultrasound guided fine needle aspiration

## References

[1] Siegel RL, Miller KD, Jemal A (2016). Cancer statistics, 2016. CA Cancer J Clin.

[2] Alzahrani AS, Alomar H, Alzahrani N. Thyroid cancer in Saudi Arabia: a histopathological and outcome study. Int J Endocrinol. 2017;2017:8423147. 10.1155/2017/8423147. Epub 2017 Feb 27. 10.1155/2017/8423147 PMC5350345 28348588

[3] Guth S, Theune U, Aberle J (2009). Very high prevalence of thyroid nodules detected by high frequency (13 MHz) ultrasound examination. Eur J Clin Invest.

[4] Al-Zaher N, Al-Salam S, El Teraifi H (2008). Thyroid carcinoma in the United Arab Emirates: perspectives and experience of a tertiary care hospital. Hematol Oncol Stem Cell Ther.

[5] Santos JE, Kalk WJ, Freitas M (2015). Iodine deficiency and thyroid nodular pathology--epidemiological and cancer characteristics in different populations: Portugal and South Africa. BMC Res Notes.

[6] Brito JP, Yarur AJ, Prokop LJ (2013). Prevalence of thyroid cancer in multinodular goiter versus single nodule: a systematic review and meta-analysis. Thyroid.

[7] Mohammadi M, et al. Iodine deficiency status in the WHO eastern Mediterranean region: a systematic review. Environ Geochem Health. 2018;40(1):87-97. 10.1007/s10653-017-9911-z. Epub 2017 Feb 21. 10.1007/s10653-017-9911-z 28224254

[8] Cooper D (2009). Revised American Thyroid Association management guidelines for adult patients with thyroid nodules and differentiated thyroid cancer: the American Thyroid Association guidelines task force on thyroid nodules and differentiated thyroid cancer. Thyroid.

[9] Cibas E, Ali S. The Bethesda system for reporting thyroid cytopathology. Thyroid. 2009;19(11):1159-65. 10.1089/thy.2009.0274. 10.1089/thy.2009.0274 19888858

[10] Al-Salamah SM, Khalid K, Bismar HA (2002). Incidence of differentiated cancer in nodular goiter. Saudi Med J.

[11] Lin JD, Chao TC, Huang BY (2005). Thyroid cancer in the thyroid nodules evaluated by ultrasonography and fine-needle aspiration cytology. Thyroid.

[12] Werk EE, Vernon BM, Gonzalez JJ (1984). Cancer in thyroid nodules. A community hospital survey. Arch Intern Med.

[13] Hadjisavva I (2015). Prevalence of Cancer in patients with thyroid nodules in the island of Cyprus: predictive value of ultrasound features and thyroid autoimmune status. Eur Thyroid J.

[14] Belfiore A, Giuffrida D, La Rosa GL (1989). High frequency of cancer in cold thyroid nodules occurring at young age. Acta Endocrinol.

[15] Harding KB, Peña-Rosas JP, Webster AC, et al. Iodine supplementation for women during the preconception, pregnancy and postpartum period. Cochrane Database Syst Rev. 2017;3:CD011761. 10.1002/14651858.CD011761.pub2. 10.1002/14651858.CD011761.pub2 PMC6464647 28260263

[16] Hussain F, Iqbal S, Mehmood A (2013). Incidence of thyroid cancer in the Kingdom of Saudi Arabia, 2000-2010. Hematol Oncol Stem Cell Ther.

[17] Laudico AV, Mirasol-Lumague MR, Mapua CA (2010). Cancer incidence and survival in metro Manila and Rizal province, Philippines. Jpn J Clin Oncol.

[18] Khan F, Ruterbusch JJ, Gomez SL (2013). Differences in the cancer burden among foreign-born and US-born Arab Americans living in metropolitan Detroit. Cancer Causes Control.

[19] Vaccarella S, Franceschi S, Bray F (2016). Worldwide thyroid-cancer epidemic? The increasing impact of overdiagnosis. N Engl J Med.

[20] Smith-Bindman R, Lebda P, Feldstein VA (2013). Risk of thyroid cancer based on thyroid ultrasound imaging characteristics: results of a population-based study. JAMA Intern Med.

[21] Cappelli C, Castellano M, Pirola I (2007). The predictive value of ultrasound findings in the management of thyroid nodules. Q J Med.

[22] Haugen BR, Alexander EK, Bible KC (2016). 2015 American Thyroid Association management guidelines for adult patients with thyroid nodules and differentiated thyroid cancer: the American Thyroid Association guidelines task force on thyroid nodules and differentiated thyroid cancer. Thyroid.

